# N-methyl-D-aspartate receptor hypofunction as a potential contributor to the progression and manifestation of many neurological disorders

**DOI:** 10.3389/fnmol.2023.1174738

**Published:** 2023-06-15

**Authors:** Bin Dong, Yang Yue, Han Dong, Yuehui Wang

**Affiliations:** ^1^Department of Geriatrics, Jilin Geriatrics Clinical Research Center, The First Hospital of Jilin University, Changchun, China; ^2^School of Psychology, Northeast Normal University, Changchun, China

**Keywords:** N-methyl-D-aspartate (NMDA) receptors, schizophrenia, GRIN related disease, NMDAR antibody encephalitis, age-related cognitive decline

## Abstract

N-methyl-D-aspartate receptors (NMDA) are glutamate-gated ion channels critical for synaptic transmission and plasticity. A slight variation of NMDAR expression and function can result in devastating consequences, and both hyperactivation and hypoactivation of NMDARs are detrimental to neural function. Compared to NMDAR hyperfunction, NMDAR hypofunction is widely implicated in many neurological disorders, such as intellectual disability, autism, schizophrenia, and age-related cognitive decline. Additionally, NMDAR hypofunction is associated with the progression and manifestation of these diseases. Here, we review the underlying mechanisms of NMDAR hypofunction in the progression of these neurological disorders and highlight that targeting NMDAR hypofunction is a promising therapeutic intervention in some neurological disorders.

## 1. Introduction

**Figure 1:**
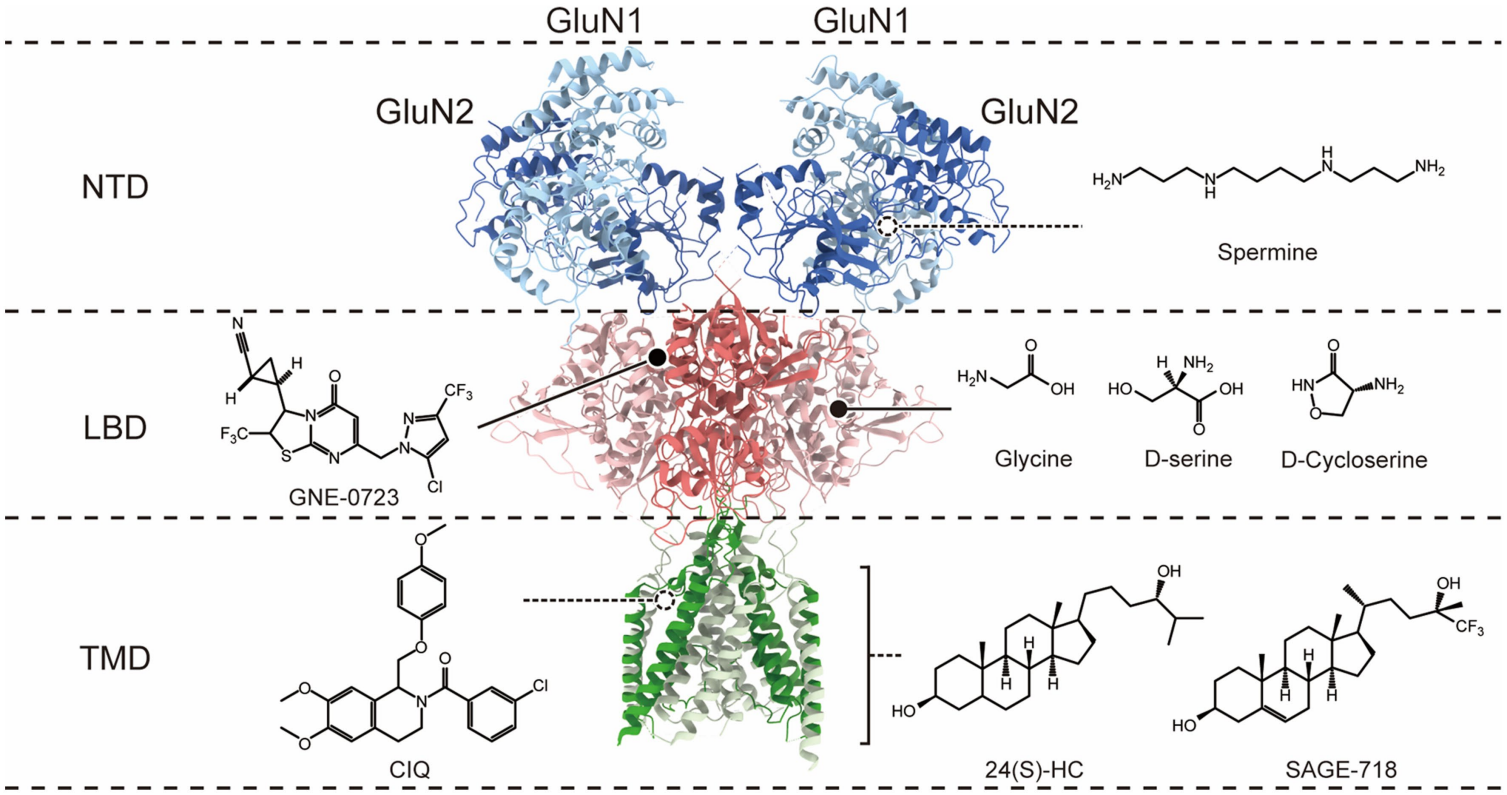
Co-agonist and allosteric modulatory binding sites on the N-methyl-D-aspartate (NMDA) receptor. The molecular structure of NMDARs is depicted, with only a dimer of GluN1 and GluN2 subunits shown for clarity. The receptor exhibits a layered architecture, with the N-terminal domains (NTDs) positioned at the top, the ligand-binding domains (LBDs) that bind glycine (Gly; or D-serine) in GluN1 and glutamate (Glu) in GluN2, the transmembrane domain (TMD), and the intracellular carboxyl-terminal domain is not present in the structure. The different domains corresponding to the binding sites of co-agonists and PAMs on the receptor are distinguished on the right.

N-methyl-D-aspartate receptors (NMDARs) are glutamate-gated ion channels permeable to Ca2+ found throughout the brain. Glutamate is the principal excitatory neurotransmitter by binding to postsynaptic NMDARs, contributing to NMDAR excitatory postsynaptic currents (EPSCs), and participating in almost all brain physiological functions. In addition, NMDAR has been implicated in synaptic plasticity, which is the key substrate for learning and memory processes (Traynelis et al., 2010; Paoletti et al., 2013). The NMDA receptor is a heterotetramer composed of four subunits, forming ion channel pores in the middle (Hansen et al., 2018). Functional NMDA receptors consist of two GluN1 subunits and two GluN2 subunits, or two GluN1 subunits, one GluN2 subunit and one GluN3 subunit (Traynelis et al., 2010). While the GluN1 subunit is encoded by GRIN1, which has eight splice variants (GluN1-1a, GluN1-1b, GluN1-2a, GluN1-2b, GluN1-3a, GluN1-3b, GluN1-4a, and GluN1-4b), the GluN2 subunit has four different isoforms (GluN2A–GluN2D) encoded by four different genes (GRIN2A–GRIN2D), and GluN3 subunit has two distinct isoforms, GluN3A and GluN3B, encoded by GRIN3A and GRIN3B. Each subunit has four similar architectural domains (Figure 1): an extracellular N-terminal domain (NTD), an extracellular ligand-binding domain (LBD) binding glutamate in GluN2 subunits, and the co-agonist glycine in GluN1 and GluN3 subunits; a pore-forming transmembrane domain (TMD); and a cytoplasmic carboxyl-terminal domain (CTD). Distinct selective features are associated with each of these different subunit domains (Swanger et al., 2016; Ogden et al., 2017; Li et al., 2019), so different isoforms of subunits combine to produce multiple NMDA receptor isoforms with unique functions. In order to activate NMDAR, it is necessary to bind both glutamate and glycine co-agonists, remove voltage-dependent ion channel blockage by Mg2+, and then open the receptor-gated ion channel, resulting in the influx of Ca2+ from the extracellular space into neurons (Hansen et al., 2018). NMDA receptor-gated Ca^2+^ influx regulates several intracellular enzymes and signaling processes critical to synaptic plasticity (Dingledine et al., 1999; Lynch, 2004; Traynelis et al., 2010; Huganir and Nicoll, 2013).

N-methyl-D-aspartate receptor dysfunction is implicated in the pathophysiology of many neurologic and psychiatric disorders. Hyperactivation of NMDARs, as observed in cerebral ischemia, brain injury, epilepsy, Huntington’s disease, and Parkinson’s disease (Mony et al., 2009; Parsons and Raymond, 2014; Wang et al., 2020), involves a process of excessive calcium influx and causing excitotoxicity (Rothman and Olney, 1995; Traynelis et al., 2010; Paoletti et al., 2013). Accordingly, most recent studies focus on NMDAR hyperfunction-associated diseases and NMDAR antagonists. However, the hypofunction of NMDARs can also have detrimental effects, such as in the pathophysiology of schizophrenia (SZ), where reduced NMDAR function is critically important. The deleterious effects of NMDAR hypofunction were confirmed by observations that the administration of noncompetitive NMDAR antagonists, phencyclidine (PCP), MK-801 and ketamine (Javitt and Zukin, 1991; Krystal et al., 1994; Coyle et al., 2003), by healthy volunteers could induce all three symptoms similar to SZ (positive, negative, and cognitive; Olney and Farber, 1995; Olney et al., 1999). SZ patients suffer worse symptoms when exposed to these compounds (Javitt and Zukin, 1991; Beck et al., 2020). Moreover, in vertebrate models, psychotic symptoms can be provoked by administrating NMDAR antagonists or genetic decline in NMDAR function (Eyer and Oghaddam, 2002; Jones et al., 2011). NMDAR hypofunction is also identified in GRIN-related diseases, especially loss-of-function genetic mutations, which make up a large part of the genetic mutation in the GRIN genes. The-loss-of function GRIN variants cause NMDAR hypofunction by various mechanisms, including reduced glutamate/glycine potency, accelerated deactivation time course, decreased surface expression, reduced channel open probability, or both. Ultimately, these variants are associated with many neurodevelopmental disorders, including epilepsy, developmental delay, autism spectrum disorder (ASD), intellectual disability (ID), and SZ (Yuan et al., 2015; XiangWei et al., 2018; Myers et al., 2019; García-Recio et al., 2021). Additionally, anti-NMDAR encephalitis is an NMDAR hypofunction-related disease that can cause several neurological symptoms, such as psychosis, seizures, abnormal behaviors, and cognitive manifestations (Lynch et al., 2018; Dalmau et al., 2019). Finally, age-related changes in cognitive performance are associated with NMDAR hypofunction (Clayton et al., 2002; Foster, 2007; Mostany et al., 2013; Kumar and Foster, 2019).

N-methyl-D-aspartate receptor hypofunction is a convergence point of pathological processes in many diseases; therefore, enhancing NMDAR function is highly therapeutically relevant. Herein, we discuss the recent research progress of NMDAR hypofunction-related diseases and pharmacological agents, hoping to provide more ideas for the research of NMDAR hypofunction-related neurological disorders.

## 2. NMDAR hypofunction as a convergence point in SZ

Schizophrenia is a progressive neurodevelopmental disorder that affects the brain before its clinical symptoms appear. Despite different neurochemical pathways and mechanisms that contribute to SZ, such as glutamatergic, GABAergic, and dopaminergic systems, the precise etiology of SZ remains unknown. Over the past few decades, the dopamine (DA) model dominated the pathophysiology hypothesis of SZ. Almost all current medications for SZ primarily work by blocking dopamine D2 receptors in the nigrostriatal pathway (Gray and Roth, 2007). Conversely, current therapeutics targeting the dopamine system have had limited success, especially in managing negative and cognitive symptoms of SZ (Uno et al., 2019; McCutcheon et al., 2020). Furthermore, most functional and structural changes of SZ patients present in the early phases of postnatal development, particularly during adolescence and juvenile years, in contrast to the emergence of dopamine dysfunction during late adolescence (Snyder and Gao, 2013; Selemon and Zecevic, 2015; Tagliabue et al., 2017; Chini and Hanganu-Opatz, 2021). Consequently, on its own, dopaminergic dysfunction cannot fully explain the wide range of symptoms and neurological deficits in SZ. The paradigm of studying schizophrenia has beyond the DA hypothesis. A major shift in the understanding of the etiology of schizophrenia proposes that multiple genetic and environmental risk factors converge on the glutamatergic system mediated by NMDARs, leading to NMDAR hypofunction in the cortical circuitry during neurodevelopment. NMDARs are widely considered critical for synaptic plasticity and circuit formation during early and late phases of neural development (Monyer et al., 1994; Sheng et al., 1994; Quinlan et al., 1999; Wang et al., 2008; Roberts et al., 2009; Wang and Gao, 2009; Snyder et al., 2013; Monaco et al., 2015). Many studies indicate that the maturation of brain circuits usually coincides with NMDAR subtype switching (e.g., from GluN2B to GluN2A and GluN3A to GluN3B), which occurs at the beginning of critical developmental periods (Monyer et al., 1994; Sheng et al., 1994; Quinlan et al., 1999; Wang et al., 2008; Roberts et al., 2009; Snyder et al., 2013). Therefore, NMDAR subtype switching renders NMDARs vulnerable to genetic and environmental risk factors (Spear, 2000). A glutamatergic model mediated by NMDARs can provide an alternative way of conceptualizing schizophrenia-associated brain abnormalities (Harrison and Weinberger, 2005; Lewis and Moghaddam, 2006; Lisman et al., 2008).

N-methyl-D-aspartate receptor hypofunction is a convergence point for the progression and symptoms of SZ, especially cognitive deficits (Moghaddam, 2003; Coyle, 2006; Lisman et al., 2008; Marek, 2010; Snyder et al., 2013; Cohen et al., 2015; Nakazawa et al., 2017). Hypofunction of NMDAR induces progressive symptoms of SZ, such as cognitive impairment and psychosis, by intervening in local deficits of gamma-aminobutyric acid (GABA; Snyder and Gao, 2013; Cohen et al., 2015; Nakazawa et al., 2017; Datta and Arnsten, 2018) and long-range disconnections between regions of the brain (Howes and Kapur, 2009; Grace, 2016). The NMDAR hypofunction hypothesis of SZ is based on observations that non-competitive NMDAR antagonists [phencyclidine (PCP), MK-801, and ketamine; Javitt and Zukin, 1991; Krystal et al., 1994; Coyle et al., 2003] can induce all three SZ-like symptoms (positive, negative, and cognitive) in healthy individuals (Olney and Farber, 1995; Olney et al., 1999). Patients with SZ patients suffer worse symptoms when exposed to these compounds (Javitt and Zukin, 1991; Beck et al., 2020). Most non-competitive antagonists work as open channel blockers of NMDAR channels (Lodge and Mercier, 2015) and directly block NMDAR channels during the depolarization phase. Therefore, NMDAR antagonists inhibit the reactivation of NMDA receptors, and synaptic transmission is disrupted concomitantly. Accordingly, the hypofunction of NMDAR is regarded to be associated with SZ-related neurocognitive symptoms.

The γ-aminobutyric acid-ergic (GABAergic) interneurons are the major inhibitory system in the brain and play a critical role in the pathological process of SZ (Lisman et al., 2008; Nakazawa et al., 2012). NMDAR antagonists mainly target constantly depolarizing neurons, especially parvalbumin fast-spiking GABAergic interneurons (Homayoun and Moghaddam, 2007). Administering antagonists to NMDA receptors causes a reduction in excitatory glutamatergic inputs to fast-spiking GABAergic interneurons, resulting in the disinhibition of pyramidal neurons and hyperexcitability in pyramidal neurons (Olney et al., 1991; Moghaddam et al., 1997; Jentsch et al., 1999; Benes, 2000; Homayoun and Moghaddam, 2007; Lisman et al., 2008). Consequently, the process evokes SZ-related phenotypes and neurochemical changes in animal models (Lindsley et al., 2006; Rujescu et al., 2006; Carpenter and Koenig, 2008; Gunduz-Bruce, 2009). For example, MK-801 displays higher affinity and specificity for NMDAR than other noncompetitive antagonists (Koek et al., 1988; Kovacic and Somanathan, 2010). MK-801 could induce SZ-like symptoms in adult rodents, including increased locomotor activity and reduced prepulse inhibition (Zhou et al., 2018). The GluN2A subunit of NMDA receptor is a faster target of MK-801 than other NMDA receptor subunits (Gielen et al., 2009), and GluN2A subunits are highly distributed in fast-spiking GABAergic interneurons (Kinney et al., 2006; Xi et al., 2009). MK-801 exhibits cell type-specific effects, blocking presynaptic NMDA receptors on axon terminals of fast-spiking neurons rather than pyramidal neurons. After 5 consecutive days of MK-801 administration in adolescent rats, blocking presynaptic NMDA receptors on excitatory inputs to fast-spiking neurons significantly reduces the amplitude of α-amino-3-hydroxy-5-methyl-4-isoxazolepropionic acid receptor (AMPAR)-mediated miniature excitatory postsynaptic currents (mEPSCs), while the frequency and amplitude of AMPAR-mEPSCs in pyramidal neurons significantly increase (Wang and Gao, 2012). The excitation-inhibition balance is changed due to reduced excitatory drive in fast-spiking interneurons and increased excitation in pyramidal neurons, resulting in excessive glutamate release, excitotoxicity, and overexcitation of pyramidal neurons (Olney and Farber, 1995; Lisman et al., 2008). Consequently, NMDAR hypofunction may cause dysfunction of the GABAergic circuitry and interruption of input and output connections between neurons, which may contribute to the major clinical characteristics of SZ.

Moreover, transgenic mice with NMDAR hypofunction displayed SZ-related phenotypes (Mohn et al., 1999). According to recent genetic studies, the mutation of the NMDAR subunit has been implicated in various neurological disorders, including SZ. However, 25 out of 370 Japanese SZ patients with rare mutations in six GRIN subunits (except GRIN2B) were identified through exome sequencing, indicating an estimated 7% of patients with idiopathic SZ carry mutations in the gene for the GRIN subunit (Yu et al., 2018). Accordingly, GRIN gene variants are relatively rare and unlikely to explain most SZ cases. Rather, there remain several high-risk genes that contribute to the hypofunction of NMDAR to increase the susceptibility to SZ, including neuregulin 1 (NRG1; Roy et al., 2007; Mei and Xiong, 2008; Kato et al., 2011), disrupted in schizophrenia-1 (DISC-1; Lipina et al., 2010; Niwa et al., 2010), and dystrobrevin-binding protein 1 (dysbindin-1; Ji et al., 2009; Papaleo and Weinberger, 2011; Papaleo et al., 2012). Neuregulin (NRG) is an SZ risk gene that signals via the receptor tyrosine kinase ErbB4 (Hahn et al., 2006). NRG1-ErbB4 signaling may reduce NMDAR-mediated currents in interneurons (Kotzadimitriou et al., 2018), contributing to the pathophysiology of SZ. NRG2 promotes ErbB4 association with GluN2B-containing NMDA receptors, followed by rapid internalization of surface receptors and potent downregulation of NMDA but not AMPA receptor currents (Vullhorst et al., 2015). Disrupted-In-Schizophrenia 1 (DISC1) is a genetic risk factor implicated in major mental disorders and plays an important role in synaptic and dendritic development in neurons (Brandon et al., 2009). DISC1 binds to and stabilizes serine racemase (SR) to regulate D-serine (an endogenous NMDAR co-agonist) production (Ma et al., 2013) and contributes to NMDAR activation. Cellular knockdown of DISC1 significantly increased NMDAR currents and NMDAR subunit expression through a phosphodiesterase 4 (PDE4)/protein kinase A (PKA)/cAMP response element-binding protein (CREB)-dependent mechanism (Wei et al., 2014), providing a potential molecular basis of DISC1 in influencing NMDAR-dependent cognitive and emotional processes. Furthermore, dysbindin-1 is highly expressed in the axon terminals of glutamatergic pyramidal neurons (Talbot et al., 2004), and it is likely to play a role in vesicular protein trafficking and synaptic transmission to influence glutamatergic function (Chen et al., 2008; Jentsch et al., 2009). The reduction of dysbindin reduces glutamate release, resulting in the dysfunction of NMDARs. Dysbindin-induced degradation of GluN1 is associated with impairments in spatial working memory (Numakawa et al., 2004; Chen et al., 2008; Jentsch et al., 2009; Karlsgodt et al., 2011). Furthermore, deficiencies in dysbindin-1 result in significant reductions in NMDAR-mediated synaptic potentiation and impaired synaptic plasticity and play a role in the cognitive impairments of SZ (Glen et al., 2014). Consequently, there is considerable evidence that SZ susceptibility genes are linked to glutamate receptors, specifically NMDARs.

*In vivo*, studies using PET imaging and single-photon emission tomography (SPECT) have revealed that the distribution volume ratio of NMDAR in the hippocampus of SZ patients was significantly lower than that of healthy controls (Pilowsky et al., 2006; Beck et al., 2021). Additionally, postmortem studies have shown that SZ patients had decreased NMDAR density and lower GluN1 mRNA and protein levels in their hippocampus than healthy controls (Vrajová et al., 2010; Rubio et al., 2012). Furthermore, results from postmortem brain studies measuring the expression of NMDAR subunits showed that in 49–73% of the GABAergic neurons in the prefrontal cortex (Bitanihirwe et al., 2009) and anterior cingulate cortex (Woo et al., 2004) of SZ patients, GluN2A mRNA expression was decreased to a level that it can no longer be detected experimentally. Moreover, a meta-analysis reported a reduction in GluN1 mRNA and protein levels in the prefrontal cortex in SZ people compared to controls (Catts et al., 2016).

Based on these observations, the hypothesis of hypofunction of NMDAR makes sense as a plausible theory for SZ (Stahl, 2007), and many researchers accept the dysfunction of NMDAR as a convergence point for SZ (Kantrowitz and Javitt, 2010).

## 3. NMDAR hypofunction is prevalent in GRIN-related disease

N-methyl-D-aspartate receptor hypofunction also is identified in GRIN-related diseases, mainly focusing on loss-of-function variants. As next-generation sequencing becomes more prevalent, numerous genetic studies have reported genetic associations between GRIN gene variants and neurodevelopmental disorders, especially rare pediatric encephalopathies. GRIN disease-associated variants affect NMDAR function by various mechanisms involving glutamate/glycine potency, course of deactivation time, surface expression, and/or open probability, ultimately resulting in neurodevelopmental disorders, including epilepsy, developmental delay, ASD, ID, and SZ (XiangWei et al., 2018; García-Recio et al., 2021). Many of these are loss-of-function variants, which could reduce overall charge transfer associated with NMDAR activation and drive some initial aspects of clinical phenotypes.

The NMDA receptor is a heterotetramer composed of two indispensable GluN1 subunits and two GluN2 and/or GluN3 subunits (Traynelis et al., 2010). Unlike GluN2 and GluN3 subunits, the presence of the GluN1 subunit is invariable and exerts a pivotal role in the normal function of NMDAR (Forrest et al., 1994; Li et al., 1994). Concomitantly, mutations affecting the GluN1 subunit could impair glutamatergic circuitry and cause severe neurodevelopmental alterations. With pathogenic GRIN1 variants first diagnosed in two patients with intellectual disability in 2011 (Hamdan et al., 2011), pathogenic GRIN1 variants have been identified in many neurological disorders. For example, loss-of-function variants of GRIN1 have been found in many patients with SZ (Begni et al., 2003; Galehdari et al., 2009; Yuan et al., 2015; Yu et al., 2018), and most epilepsy-related GRIN 1 variants are loss-of-function (D552E, Q556*, S560dup, Y647S, G815R, and G827R; Lemke et al., 2016; Xu and Luo, 2018). Meanwhile, mutation scattered along the M4-CTD region and the ATD induces a dramatic decline in NMDAR surface expression, generating a loss-of-function effect and clinical symptoms (Santos-Gómez et al., 2021b). A vitro study using GRIN 1−/− knockdown mice (GluN1KD) found that loss-of-function GRIN 1 mutations reduce the volume of dopaminergic structures in the early stages of development. In contrast, delayed changes in the structure of limbic and white matter of the brain are more evident after puberty (Intson et al., 2019). Furthermore, smaller brain volume has been reported in patients with GRIN1 variants with loss of function related to encephalopathy and SZ (Brown et al., 1986; Cahn et al., 2002; van Haren et al., 2008; Lemke et al., 2016; Rossi et al., 2017; Fry et al., 2018).

The GluN2 subunit genes, especially GRIN2A and GRIN2B (which encode GluN2A and GluN2B subunits, respectively), are highly intolerant to genetic variations, and the incidence of *de novo* genetic variations is high (Higley and Sabatini, 2012; Otsu et al., 2019). Mutations in these genes were generally correlated with NMDAR-related neurodevelopment disorders, with 43 and 35% of NMDAR mutations, respectively (Hansen et al., 2021).

An in-depth investigation of GRIN2A-related phenotypes, encompassing 248 affected individuals with pathogenic or probable pathogenic variants in GRIN2A, suggested that missense variants, like C436R, G483R, V685G, P699S, M705V, P716T, A727T, and D731N, within amino-terminational or ligand-binding domains exclusively caused NMDAR loss-of-function (Lemke et al., 2013; Lesca et al., 2013; Swanger et al., 2016; Addis et al., 2017; Strehlow et al., 2019). Most rare variants of GluN2A were associated with epilepsy and ID. The GluN2A(D731N) variant, which resides in a section of the LBD, could decrease glutamate potency, channel open probability, charge transfer, and receptor surface expression (Gao et al., 2017). The GluN2A (C436R) variant, which also resides in LBD, impairs the folding and stability of protein structure by disrupting the disulfide bond formation (Elmasri et al., 2022). Additionally, GRIN2A and GRIN2B pathogenic protein truncating variants lead to the reduction or total loss of NMDARs function, as variant products are insufficient to access the cell surface, resulting in a significant reduction of NMDAR-mediated currents (Santos-Gómez et al., 2021a; De Bernardi et al., 2022). Each of these GRIN2 mutations results in a loss-of-function effect on NMDARs.

While homozygous mice with GRIN 2B deletion showed a long-term depression impairment in the hippocampal area and died with a deficiency of suckling response, heterozygous mice survived with decreased NMDAR surface expression (Kutsuwada et al., 1996). It has been identified that loss-of-function mutations in GRIN2B are associated with ID and ASD (Endele et al., 2010; O'Roak et al., 2011). Some missense genetic variants of GRIN2B can damage GluN2B function and cause loss of function. For example, GluN2B (E413G and C461F), located in the agonist-binding domain, reduces Glu potency, current density, and deactivation time (Adams et al., 2014; Swanger et al., 2016; Bell et al., 2018; Fedele et al., 2018; Wells et al., 2018). GluN2B S541R, located in linker regions, reduces Glu potency (Platzer et al., 2017). GluN2B (E413G, C436R, and C456Y) revealed decreased surface expression (O'Roak et al., 2012; Adams et al., 2014; Swanger et al., 2016; Platzer et al., 2017; Bell et al., 2018; Fedele et al., 2018; Wells et al., 2018). Additionally, analyses of GRIN2B protein truncation variants revealed decreased NMDAR-mediated currents and surface expression, eventually leading to NMDAR functional haploinsufficiency (Sceniak et al., 2019; Santos-Gómez et al., 2021a). Interestingly, using clonal models of loss of function mutations and electrophysiology and calcium imaging method, Bell et al. (2018) demonstrated that loss of GRIN2B function might be in a more proliferative-like state, altering differentiation and, presumably, how neurons integrate into developing circuits.

## 4. NMDAR hypofunction induced by internalization of receptors in anti-NMDAR encephalitis

Anti-NMDAR encephalitis is a severe neuropsychiatric disorder, considered a NMDAR hypofunction-related disease. It is responsible for various neurological symptoms such as psychosis, seizures, abnormal behaviors, and cognitive manifestations (Lynch et al., 2018; Dalmau et al., 2019). NMDAR internalization causes NMDAR hypofunction in anti-NMDAR encephalitis, a process where antibodies crosslink NMDARs on the surface of neurons, resulting in a reversible reduction of NMDARs on the surface (Hughes et al., 2010; Mikasova et al., 2012; Moscato et al., 2014; Jézéquel et al., 2018). Molecular studies about the antibody binding sites identified that antibodies recognize the epitope on NTD of GluN1 in anti-NMDAR encephalitis, which contains N368/G369 (Dalmau et al., 2008; Gleichman et al., 2012; Kreye et al., 2016; Sharma et al., 2018; Jones et al., 2019). Antibody-binding epitopes overlap or be adjacent to the GluN1-EphB2-interacting sites on GluN1 (Mikasova et al., 2012), and the NMDAR-EphB2 interaction contributes to the stability of NMDARs in synapses (Dalva et al., 2000). Therefore, NMDAR antibodies could disrupt the interaction between NMDARs and EphB2 receptors, and then alter the localization of NMDARs accompanied by internalization (Mikasova et al., 2012). Although studies with super-resolution and single molecule localization microscopy have shown that treatment with NMDAR antibodies in cultured neurons induces a reduction of NMDARs on the surface and diffusion of remaining receptors (Ladépêche et al., 2018), it does not affect the ionotropic function of NMDAR channels before receptor internalization. Treatment with autoantibody-containing cerebrospinal fluid (CSF) in patients does not reduce NMDAR-mediated currents within 30 min (Mikasova et al., 2012; Moscato et al., 2014), significantly reduces them after 24 h due to receptor internalization/lateral diffusion (Hughes et al., 2010).

Research utilizing passive transfer models has provided positive evidence that the chronic transfer of patient antibodies leads to a decrease in endogenous NMDARs in synapses, a reduction in NMDAR-mediated currents, impaired NMDAR-dependent LTP, compromised spatial memory and novel object recognition, depression-like behaviors, and seizures. Mice treated with autoantibody-containing CSF of patients exhibited impaired LTP in Schaffer collateral-CA1 synapses and decreased NMDAR densities and EphB2 expression (Planagumà et al., 2016). Meanwhile, hippocampal CA1 and CA3 also observed the impairment of NMDAR-dependent LTP in female Wistar rats with the infusion of autoantibody-containing CSF of patients (Blome et al., 2018; Kersten et al., 2019). Additionally, various studies have reported that patient’s NMDAR antibodies consistently decrease the novel object discrimination index in mice and rats after the passive transfer of patient antibodies, indicating a decline in short-term/working memory (Planagumà et al., 2015, 2016; Malviya et al., 2017; Kersten et al., 2019; Carceles-Cordon et al., 2020). Cortical inhibitory neurons—GABAergic interneurons are the main target of hNR1 antibodies (a class of human monoclonal antibodies targeting the GluN1 subunit; Kreye et al., 2016; Andrzejak et al., 2022). With the internalization and hypofunction of NMDARs in GABAergic interneurons, the inhibitory drive onto excitatory neurons dramatically decreases (Nakazawa et al., 2017), which may explain the psychosis symptom of anti-NMDAR encephalitis. Furthermore, Wright et al. (2021) found that the decrease in excitatory neurotransmitter transport caused by NMDAR antibodies may be the cause of seizures in anti-NMDAR encephalitis patients.

Ovarian teratomas and herpes simplex virus (HSE) infections may be the main causes of anti-NMDAR encephalitis. The clinical manifestations of anti-NMDAR encephalitis are consistent with the results of *in vitro* and animal experiments. It is widely accepted that NMDAR autoimmune antibodies are responsible for encephalitis, as they crosslink NMDARs, disrupt NMDAR-EphB2 interactions, and cause NMDAR internalization. This internalization leads to NMDAR hypofunction, which is believed to be the cause of impaired long-term potentiation, memory, and behavioral deficits, and increased susceptibility to seizures. Once diagnosed, immunotherapy should be given actively, and antibodies can be neutralized and degraded indirectly. In addition, tumor removal should be performed as soon as possible for patients with associated tumors. The above treatments can accelerate symptom recovery and reduce the risk of recurrence.

## 5. NMDAR hypofunction is associated with age-related cognitive decline

Normal aging, a natural process characterized by structural and functional changes in the brain, leads to cognitive decline, mainly in learning and memory performance. Age-related cognitive performance is associated with hypofunction of NMDAR (Clayton et al., 2002; Foster, 2007; Mostany et al., 2013; Kumar and Foster, 2019). The NMDAR plays a crucial role in cognitive performance, particularly in learning and memory performance (Kuehl-Kovarik et al., 2000; Paoletti and Neyton, 2007; Forsyth et al., 2015). The expression of NMDA receptor-associated signaling molecules attenuates with increasing age (Gonzales et al., 1991; Pittaluga et al., 1993).

Cognitive declines are related to changes in the specific subunits in NMDARs with advancing age. The GluN2B subunit plays a major role in forming and maintaining learning and memory (Sakimura et al., 1995; Malenka, 2003; Zhuo, 2008). The expression of GluN2B subunits decreased significantly than the other subunits in the forebrain of aged rodents, especially in the cerebral cortex and hippocampus (Magnusson, 2001; Ontl et al., 2004; Magnusson et al., 2006; Zhao et al., 2009). This reduction induced a decrease in the transmission of GluN2B-dependent signaling molecules that contribute to long-term potentiation and synaptic plasticity (Hardingham and Bading, 2010; Lussier et al., 2015), followed by decreased NMDAR function and NMDAR-related cognitive decline (Clayton and Browning, 2001; Magnusson et al., 2007; Zhao et al., 2009). Further analyses of human temporal cortical tissue obtained during neurosurgery declared a progressive reduction in GluN2B subunits in the aging human brain (Pegasiou et al., 2020). This indicates that NMDARs containing GluN2B subunits exert a critical role in synaptic responses in the adult human brain, but their influence is diminished in the elderly. NMDAR hypofunction is also implicated in Alzheimer’s disease (AD). Significant reductions in the expression of the GluN2B subunit have also been seen in the hippocampus and the entorhinal cortex of brains with AD (Sze et al., 2001), suggesting a relationship between GluN2B-related NMDAR hypofunction and AD. Furthermore, upregulating GluN2B expression in the brain by dephosphorylating (Plattner et al., 2014), protein kinase inhibitors (Hawasli et al., 2007), or genetic overexpression (Wong et al., 2002; Cao et al., 2007; Yin et al., 2011, 2012) resulted in significant changes in learning and memory in aged rodents. These data suggest that the GluN2B subunit is an ideal target for improving age-related cognitive decline.

Although many previous works supported the viewpoint that the expression of the GluN2B subunit decreased with age and contributed to the decline of cognitive function in the elderly, there are still some studies that proposed different views. Proximity ligation assay (PLA) analyses revealed disrupted GluN2B-containing NMDAR trafficking in aged mice, resulting in synaptic accumulation of these receptors in the apical dendrites of dCA1 (Zamzow et al., 2013; Al Abed et al., 2020). In the hippocampal region, GluN2B association with membrane scaffolding proteins, including PSD95, augments with age (Zamzow et al., 2013). These all resulted in hypofunction of NMDARs, followed by age-related memory impairment. Interestingly, some researchers examined the role of the GluN2A and GluN2B subunits in the hypofunction of the NMDAR synaptic due to aging, indicating that the age-related decrease in response to NMDAR is not due to a change in the ratio of diheteromeric GluN2A/GluN2B subunits in the synapse. The meaningful result is that age-related oxidative stress disrupts synaptic plasticity and suppresses NMDAR function (Kumar et al., 2019).

Meanwhile, compared to the GluN2B subunit, there are conflicting results about the GluN1 expression by aging, with meaningful declines in some studies but not in others (Magnusson et al., 2005; Das and Magnusson, 2011). Age-related lower expression of the GluN1 subunit shows a relationship to the hypofunction of the NMDAR, impairing spatial reference memory performance in old rats and mice (Adams et al., 2001; Magnusson et al., 2007; Das and Magnusson, 2008). Nonetheless, a study by Zhao et al. (2009) found no significant alteration in GluN1 protein expression in the hippocampus of aged mice, while another study by Gramuntell et al. (2021) reported no variation in the density of GluN1 protein in the oriens-lacunosum molecular cells in the hippocampal CA1 region with aging.

Other factors, including hormones (Lichtenwalner et al., 2001; Li et al., 2004), changes of neuropeptides, and synaptic loss (Burke and Barnes, 2006), oxidative stress, inflammatory response, also play a critical role in age-related cognitive decline. The damage to NMDAR structure and function can be caused by these factors, which may converge to cause NMDAR dysfunction, making it a potential convergence point in the pathological process of age-related cognitive impairment. While the exact mechanisms and treatments of NMDAR dysfunction remain unclear, investigating this area is highly significant for comprehending the pathological processes of cognitive disorders and discovering novel approaches to treatment.

## 6. Reverting NMDAR hypofunction by pharmacological agents

Regarding therapeutic potential, NMDARs are highly interesting targets. Overactivation of NMDARs may have a deleterious effect, which limits the therapeutic window for enhancing NMDAR activity. This may lead to the difficulty of improving NMDAR function through direct activation *via* glutamate binding sites located in the LBD. Therefore, most of the current studies aimed at improving NMDAR hypofunction are based on endogenous co-agonists and positive allosteric modulators (PAMs).

Clinical interventions for NMDAR hypofunction mainly rely on co-agonists to increase the occupancy of glycine co-agonist binding sites (Figure 1), such as glycine, D-serine, and D-cycloserine. However, the application of high concentrations of glycine may lead to the internalization of NMDAR *in vitro* (Nong et al., 2003), so the application of glycine may ultimately reduce rather than increase NMDAR activity. D-serine is an endogenous NMDAR co-agonist that functions in a glycine environment at physiological concentrations (Harsing and Matyus, 2013). In the aging brain, the level of D-serine in the brain decreases, leading to NMDAR hypofunction. Studies have shown that supplementing D-serine in rat experiments reversed age-related cognitive flexibility and dendritic spine density decline and partially restored large-scale functional connections (Nava-Gómez et al., 2022). Another clinical study showed that administering a combined metabolic activator containing 61.75% L-serine (the endogenous D-serine precursor) orally to AD patients improved cognitive function by 29% after 84 days (Yulug et al., 2023). Furthermore, L-serine has been administered as a dietary supplement to a 5-year and 10-month-old patient diagnosed with GRIN2B-related loss of function mutation and suffering from severe encephalopathy. Encouragingly, the patient showed significant improvements in motor and cognitive function after 11 and 17 months of L-serine dietary supplementation (Soto et al., 2019), suggesting that L-serine may have a therapeutic role in treating NMDAR hypofunction. D-cycloserine (DCS), another partial agonist located at the NMDAR glycine binding site (Monahan et al., 1989), enhances receptor activation in the presence of glutamate. *In vitro*, studies have shown that DCS promoted NMDAR-dependent synaptic potentials (Billard and Rouaud, 2007), and intrahippocampal infusion of DCS enhanced the expression of the NMDAR subunit GluN2B in the hippocampus of young rats (Ren et al., 2013), which may contribute to enhancing long-term potentiation (LTP) and memory. In the treatment of schizophrenia, the role of co-agonists (including glycine, D-serine, and DCS) has also been extensively studied, but most of the results are contradictory or, overall, do not significantly improve negative and cognitive symptoms.

Over the past decade, NMDAR-positive allosteric modulators have gained substantial interest in counteracting diseases associated with the hypofunction of NMDARs. Allosteric modulators target allosteric sites that have high freedom and low conservatism (Figure 1), which allows for higher selectivity and avoids competition with ligands. Some allosteric modulators are relatively safe, even at high doses. The binding of allosteric modulators to specific structural domains can lead to long-range structural rearrangements that affect receptor activity. For example, conformational changes in NTDs can induce concomitant motions of the LBD and TMD, providing allosteric control of channel activity (Zhu and Paoletti, 2015; Hansen et al., 2018; Esmenjaud et al., 2019). Some of these PAMs have shown therapeutic potential in animal models or humans with NMDAR hypofunction-related diseases.

Polyamines at micromolar concentrations can selectively enhance the GluN2B subtype of NMDARs (Williams, 1994; Mony et al., 2009). Polyamine spermine binding to the lower lobe interface of GluN1 and GluN2B NTDs stabilizes this interface in a tight conformation, possibly by reducing the repellency between negative charges in the lower lobe of the NTD, which may result in a higher probability of channel opening (Mony et al., 2011; Sirrieh et al., 2015; Esmenjaud et al., 2019). The effects of polyamines have been studied in various cognitive and behavioral paradigms in animal experiments, and spermine administration has been shown to rescue learning and memory deficits in neuroinflammatory animal models and Huntington’s disease models, both of which are associated with neurodegeneration and cognitive decline (Velloso et al., 2008; Frühauf et al., 2015). However, polyamines have shown harmful effects in Alzheimer’s disease models (Guerra et al., 2016). GNE compounds are specific PAMs for GluN2A-NMDARs discovered through high-throughput screening. GNE PAMs may bind at the D1-D1 interface between GluN1 and GluN2A LBDs (Hackos et al., 2016) and enhance NMDAR activity by stabilizing the dimer interfaces between LBDs and preventing the receptor from desensitization. Chronic administration of an oral bioavailable derivative of GNE, GNE-0723, has been shown to improve learning and memory retention in AD mice in the Morris water maze task and in Dravet Syndrome mice in the contextual fear conditioning task, and the research also revealed the therapeutic potential of GNEs in conditions characterized by epileptic discharges and network overactivity (Hanson et al., 2020). CIQ is a selective PAM for GluN2C/2D-NMDARs, with no activity on other NMDAR, AMPA, kainate, GABA, or glycine receptors. The CIQ binding site is located in the outer transmembrane region and involves the pre-M1 and M3 regions of GluN2, as well as the neighboring pre-M4/M4 region of GluN1, forming the “gating triad” (Perszyk et al., 2020), and acts by disrupting the closed state of the receptor (Ogden and Traynelis, 2013; McDaniel et al., 2020). CIQ administered systemically partially alleviated abnormal sensory-motor gating, hyperactivity, and stereotypy in animals treated with MK-801 and methamphetamine, as well as improved working memory in mice treated with MK-801 (Suryavanshi et al., 2014). 24(S)-hydroxycholesterol (24(S)-HC), an endogenous compound, is the most prevalent metabolite of cholesterol in the brain. The compound has a high selectivity toward NMDARs, with little or no effect on AMPAR or GABAAR (Paul et al., 2013). 24(S)-HC exerts its activity through the TMD (Wilding et al., 2016), increases channel open probability, and enhances NMDARs function at saturating concentrations of agonist. Reduced levels of 24(S)-HC in the brain have been implicated in several neurodegenerative diseases, such as aging, AD, or Parkinson’s disease. Mice deficient in cholesterol 24-hydroxylase, an enzyme responsible for converting cholesterol to 24(S)-hydroxycholesterol, exhibited severe learning deficits and impaired CA3-CA1 synaptic LTP (Kotti et al., 2006; Russell et al., 2009). Studies have shown that 24(S)-HC or its derivatives SGE-201 and SGE-301 can increase STP and LTP in brain slices of aged rats, partially reversed behavioral deficits in rats caused by MK801 or PCP injections (a pharmacological model of SZ; Paul et al., 2013), and reversed memory loss in mice in the anti-NMDAR encephalitis model after chronic administration of SGE-301 (Mannara et al., 2020). Additionally, a 24(S)-HC derivative SAGE-718 has shown promising results in a phase 2 clinical trial (LUMINARY) for the treatment of Alzheimer’s disease (AD), improving executive function, learning, and memory in patients with mild cognitive impairment (MCI) and mild dementia, with good tolerability (Koenig et al., 2022). Although 24(S)-hydroxycholesterol (24(S)-HC) has shown great therapeutic potential in various neurological disorders, it is important to note that high concentrations of 24(S)-HC may trigger or aggravate NMDAR-induced neurotoxicity (Sun et al., 2017).

## 7. Conclusion

Herein, we provide an overview of the research progress on the neurobiological effects of NMDAR hypofunction, which is associated with many neurological and psychiatric disorders. We also discuss the research progress on pharmacological agents that directly target the gating mechanisms of NMDAR to enhance its function. We are pleased to see that there is currently a lot of research on co-agonists and PAMs that aim to improve NMDAR hypofunction, and encouraging progress has been made in various NMDAR hypofunction-related neurological diseases. In particular, PAMs have great therapeutic potential. However, there are still many issues that need to be addressed in the research on co-agonists and PAMs.

N-methyl-D-aspartate receptor PAMs can act in various ways, including increasing the maximum open channel probability of the receptor (e.g., glycine and GNE −6901), increasing the sensitivity of the receptor to glycine (or polyamines), and increasing the potency of glutamate (e.g., GNE -8304 or GNE -0723; Hackos and Hanson, 2017). Several NMDAR PAMs can effectively alleviate some behavioral and cognitive symptoms in animal models of NMDAR hypofunction. However, the physiological effects and therapeutic potential of any given NMDAR PAM usually depend on multiple factors, so it seems very difficult to predict the physiological outcomes of NMDAR PAMs based solely on the characteristics of their recombinant receptors. For example, two compounds, GNE-6901 and GNE-8314, which have the same GluN2A subunit selectivity and binding site, may have completely opposite effects on synaptic physiology (LTP) because GNE-8314 may act preferentially on interneurons (Hackos et al., 2016; Yao et al., 2018). Furthermore, due to the low conservation of allosteric sites, there are huge differences in allosteric regulation mechanisms between different species. Therefore, it is difficult to evaluate or predict the results of clinical trials of allosteric modulators based on animal experiments, and even contradictory results may be obtained. In elderly animals and humans, NMDAR expression levels and NMDAR-mediated EPSCs are all decreased, especially the expression of the GluN2B subunit. Similarly, in patients with anti-NMDAR encephalitis and mice induced with anti-NMDAR IgGs, NMDAR expression is reduced by 50–80% (depending on the titer; Mannara et al., 2020). However, when the remaining NMDARs are too few, it may be difficult to achieve significant cognitive and behavioral rescue by just enhancing the remaining NMDARs. In neurological diseases caused by NMDAR gene mutations, gene mutations may cause structural changes in allosteric binding sites, preventing allosteric modulators from binding, or may affect the signaling pathways of allosteric regulation, resulting in unknown effects. However, it is worth noting that a child with the loss-of-function mutation GluN2B-P553T showed improvement in behavioral and cognitive symptoms after treatment with L-serine (Soto et al., 2019). This clinical study serves as a compelling example of how enhancing the overall activity of NMDAR may be beneficial in rescuing the hypofunction of a specific NMDAR subtype.

The allosteric modulation mechanism may transform non-druggable targets into druggable targets, with corresponding allosteric modulators having higher selectivity and safety. This brings new opportunities for the study of NMDAR structure and function and the treatment of some NMDAR hypofunction-related neurological diseases. Furthermore, studies have proposed an unconventional NMDAR signaling (Dore et al., 2017), non-ionotropic NMDAR signaling, which is believed to arise from conformational changes in the receptor induced by agonists, independent of channel opening. This type of signaling is more likely to occur when there is a decrease in Ca2+ influx through the NMDAR, resulting in synaptic depression and reduction of spines. These changes could be a contributing factor to the reduced number of dendritic spines and cognitive impairments observed in individuals with schizophrenia. These findings may potentially pave the way for novel pharmacological interventions aimed at targeting distinct synaptic signaling pathways in neurological disorders such as schizophrenia. Due to the highly complex nature of NMDAR, the relationship between its structure and function, as well as the molecular basis of its diverse functions, warrant further research and exploration in the future.

## Author contributions

BD: conceptualization, writing—original draft. YY and HD wrote the sections of the manuscript. YW: conceptualization, resources, supervision, and writing—review and editing. All authors contributed to the article and approved the submitted version.

## Conflict of interest

The authors declare that the research was conducted in the absence of any commercial or financial relationships that could be construed as a potential conflict of interest.

## Publisher’s note

All claims expressed in this article are solely those of the authors and do not necessarily represent those of their affiliated organizations, or those of the publisher, the editors and the reviewers. Any product that may be evaluated in this article, or claim that may be made by its manufacturer, is not guaranteed or endorsed by the publisher.
